# Is There Long-Term Clinical Equipoise Between CABG and PCI for Isolated Left Anterior Descending Artery Disease?

**DOI:** 10.1016/j.jscai.2023.100611

**Published:** 2023-05-19

**Authors:** Eliano P. Navarese, Eleonora Ruscio, Diana A. Gorog

**Affiliations:** a Interventional Cardiology and Cardiovascular Medicine Research, Department of Cardiology and Internal Medicine, Nicolaus Copernicus University, Bydgoszcz, Poland; b Faculty of Medicine, University of Alberta, Edmonton, Canada; c SIRIO MEDICINE Research Network, Poland; d Department of Cardiology, Catholic University Medical School, Rome, Italy; e Faculty of Medicine, National Heart and Lung Institute, Imperial College, London, United Kingdom; f Postgraduate Medical School, University of Hertfordshire, Hatfield, United Kingdom

**Keywords:** coronary artery bypass surgery, left anterior descending coronary artery, percutaneous coronary intervention

For decades, numerous randomized controlled trials and meta-analyses have investigated comparative outcomes in patients with stable coronary artery disease—more recently termed chronic coronary syndrome—undergoing percutaneous coronary intervention (PCI) and coronary artery bypass graft surgery (CABG). Several studies have shown the superiority of CABG over PCI in patients with higher disease burden, greater lesion complexity, and in the presence of diabetes, although recent large-scale analyses at 10-year follow-up have shown substantial equivalence of the 2 strategies in patients with diabetes.^1^

Comparisons of CABG and PCI, which have demonstrated the survival benefit of CABG, have also shown a reduction in myocardial infarction (MI). The proposed reason for the lower MI rates with CABG was that PCI is a focal treatment of the individual area of atherosclerosis, whereas surgery usually bypasses longer segments of the coronary tree and therefore may be protective not only against the flow-limiting lesion that it is bypassing but also against other diffuse areas of atherosclerosis in the same vessel, including nonobstructive yet vulnerable plaques that might be prone to rupture and predispose to further MIs. Most bypass grafts are placed distally, and thus, they might be protective of the entire vulnerable region.

Another postulated mechanism to explain the possible superiority of surgery is that CABG could offer virtual collateralization. Similar to that the effect obtained through native collateralization, which can be cardioprotective against long-term MI, virtual collateralization theoretically prevents symptomatic plaque rupture and vessel occlusion^2^; however, the studies in support of this hypothesis often involved patients with multivessel disease and did not distinguish between periprocedural and spontaneous MI.^3^ In this context, conflicting data exist on whether CABG could prove superior to PCI in isolated left anterior descending artery (LAD) disease.

In this issue of *JSCAI*, Prasad et al^4^ re-evaluated this subject by performing a meta-analysis to compare PCI and CABG revascularization modalities for isolated LAD disease. The authors included long-term follow-up data from randomized studies. The primary outcome of interest was Q-wave and non–Q-wave MI (procedural and nonprocedural) at latest follow-up. Secondary end points included all-cause death and target vessel revascularization at latest follow-up. The longest follow-up from each study ranged from 4 to 10 years (weighted mean average 8.3 years). The authors should be commended for conducting this study, properly designed to address the question of optimal revascularization modality for isolated LAD disease.

Four randomized controlled trials with 573 patients were included. The incidence of MI at long-term follow-up was similar between the CABG and PCI groups (relative risk ratio [RR], 1.33; 95% CI, 0.62-2.83). Mortality did not significantly differ between the 2 strategies (RR, 1.04; 95% CI, 0.70-1.55), indicating equivalence of these strategies over long-term follow-up. On the contrary, target vessel revascularization, a surrogate end point, was reduced in the CABG group (RR, 0.27; 95% CI, 0.15-0.46).

An interesting feature of this analysis is that the authors selected the rates of spontaneous MI occurring >4 years of follow-up as end point, which allowed the authors to disentangle the MI components (spontaneous and procedural) and focus on spontaneous MI, the most clinically relevant in terms of survival. The fact that certain thresholds of periprocedural MI affect survival has led to the mistaken belief that all procedural MI types should be treated as equally prognostically important and the misinterpretation that they are comparable to spontaneous MIs. In a recent landmark meta-analysis comparing revascularization vs optimal medical therapy in stable patients, only spontaneous MI, not periprocedural MI, was found to be related to cardiac mortality.^5^ Recent analyses of periprocedural biomarker elevations have questioned the relationship between periprocedural MI and subsequent mortality following PCI. In a large patient-level meta-analysis, on multivariate analysis, a significantly increased risk for late mortality after PCI was only observed among patients who had creatine kinase–myocardial band of ≥10× the upper limit of normal^6^; however, cardiac troponin elevations were not associated with increased mortality.

The relevance of long-term follow-up to properly address the true magnitude of the effect of revascularization strategies has been discussed.^7^ The long follow-up time selected allowed the temporal accrual of MI and death rates over time in a powered fashion^7^ that, in turn, triggers considerations on the absence of a superior cardioprotective effect of CABG compared with PCI in the context of a properly designed meta-analysis.

Another compelling aspect of this meta-analysis is the low-to-absent heterogeneity among the studies for death and MI outcomes, which suggests that device/surgical iterations over time and study chronology did not seem to affect the findings of this meta-analysis.

Limitations of the report by Prasad et al should be discussed. Only data from randomized studies were analyzed, lowering the chance of spurious results due to the inclusion of observational studies, prone to confounding; however, information about lesion complexity, which might have affected outcomes of PCI vs CABG, was not routinely available. The meta-analysis included studies with bare metal stents and drug-eluting stents. Including only drug-eluting stents, however, would have led to underpowered results but presumably not altered them or slightly numerically favored the percutaneous strategy, given the use of more modern and biocompatible devices in the PCI arm.

Although currently less applicable in routine practice, a fine-tuned interrogation of these plaques with dedicated intravascular coronary imaging tools beyond sole angiography might guide the decision to revascularize the nonobstructive atherosclerotic lesions beyond the flow-liming stenosis. Such identification of vulnerable plaques with lipid-rich cores, known to be associated with adverse clinical events,^8^ might better guide operators toward the optimal revascularization strategy, whether percutaneous or surgical.

Alternatively, baseline low-density lipoprotein cholesterol (LDL-C) thresholds have been found to be a marker of the atherosclerotic burden and plaque destabilization^9^ and predictors of clinical events in recent analyses.^10^ One might postulate that patients with LDL-C thresholds >100 mL/dL would reap lower benefits from PCI compared with CABG, whereas outcomes may be similar with lower LDL-C levels. Both intravascular imaging and LDL-C threshold-based hypotheses to guide revascularization strategies need to be validated in dedicated studies in the context of isolated LAD disease.

The study by Prasad et al is a foray into this important area of the comparative efficacy of percutaneous and surgical revascularization modalities in isolated LAD lesions, showing the long-term clinical equipoise of the 2 revascularization modalities.
